# Fluorescence Quenching of Tyrosine-Ag Nanoclusters by Metal Ions: Analytical and Physicochemical Assessment

**DOI:** 10.3390/ijms23179775

**Published:** 2022-08-29

**Authors:** Ditta Ungor, Rita Bélteki, Krisztián Horváth, Orsolya Dömötör, Edit Csapó

**Affiliations:** 1 MTA-SZTE Lendület “Momentum” Noble Metal Nanostructures Research Group, University of Szeged, Rerrich B. Sqr. 1, H-6720 Szeged, Hungary; 2 Interdisciplinary Excellence Center, Department of Physical Chemistry and Materials Science, University of Szeged, Rerrich B. Sqr. 1, H-6720 Szeged, Hungary; 3 Department of Inorganic and Analytical Chemistry, University of Szeged, Dóm Sqr. 7, H-6720 Szeged, Hungary

**Keywords:** tyrosine, Ag nanocluster, metal ions, fluorescence quenching, dark complex, thermodynamic evaluation

## Abstract

A new synthesis method is described for the first time to produce silver nanoclusters (AgNCs) by using the tyrosine (Tyr) amino acid. Several important parameters (e.g., molar ratios, initial pH, reaction time etc.) were optimized to reach the highest yield. The formed Tyr-AgNCs show characteristic blue emission at λ_em_ = 410 nm, and two dominant fluorescence lifetime components were deconvoluted (τ_1_ ~ 3.7 and τ_2_ ~ 4.9 ns). The NCs contained metallic cores stabilized by dityrosine. For possible application, the interactions with several metal ions from the tap water and wastewater were investigated. Among the studied cations, four different ions (Cu^2+^, Ni^2+^, Fe^3+^, and Rh^3+^) had a dominant effect on the fluorescence of NCs. Based on the detected quenching processes, the limit of detection of the metal ions was determined. Static quenching (formation of a non-luminescent complex) was observed in all cases by temperature-dependent measurements. The calculated thermodynamic parameters showed that the interactions are spontaneous ranked in the following order of strength: Cu^2+^ > Fe^3+^ > Rh^3+^ > Ni^2+^. Based on the sign and relations of the standard enthalpy (Δ*H*°) and entropy changes (Δ*S*°), the dominant forces were also identified.

## 1. Introduction

In recent years, the development of new nanomaterials has been one of the most popular research topics in science. Noble metal nanostructures show several interesting properties depending on their composition, size and shape [1]. It is well-known that classical gold (Au), copper (Cu) and silver (Ag) nanoparticles (NPs) show localized surface plasmon resonance (LSPR) in the nanometer range size, which can be identified as a characteristic band in UV-visible spectra [2,3]. For synthesis, several approaches can be found in the literature [4], but over the last few years, green chemistry methods [5,6,7] have become of interest considering the possible biomedical applications of these nanostructures [8,9]. In these procedures, mild conditions are used to produce biocompatible and stable colloidal systems. For this purpose, a single biomolecule promotes the reduction of metal ions but also provides both stabilization and functionalization of the NPs formed [10,11].

Related to these methods, it should be noted that the applied molar ratio between the precursor metal ions and the biomolecule plays a great role in the size and optical feature of the formed nano-object [12]. If the ligand-to-metal molar ratio is small, the result of the synthesis is the above-mentioned plasmonic NPs. In contrast, if a high ligand excess is used, the formed particles have sub-nanometer average sizes. Therefore, these ultra-small particles, named nanoclusters (NCs), show unique molecular-like fluorescence. It is generally accepted that the fluorescence of Au- and AgNCs strongly depends on the number of metal atoms in the cluster cores, and the quality of the stabilizing ligands also has decisive effect on optical properties.

Several articles have studied the possible application of proteins [13,14], nucleotides [15,16,17,18,19] and simple amino acids [20,21] for the synthesis of fluorescent NCs. These molecules possess several advantageous sulfur- or nitrogen-containing functional groups to bind the soft noble metal ions in the preliminary coordinative interaction [22,23,24,25,26,27,28]. They also have great pH-dependent redox activity [29,30] to form ultra-small cores having metal atoms with predominantly zero oxidation states. Only two articles have been published previously on the application of Tyr for the production of Au NCs. X. Yang et al. presented an interesting work about the simple synthesis of Tyr-stabilized AuNCs without any additional reducing agent [31]. For their preparation, a Tyr:Au/1.8:1 molar ratio was applied under acidic conditions at 37 °C for 24 h. The formed NCs showed blue emission at 470 nm with a quantum yield (QY) of 2.5%. The size of the Au cores varied between 1–3 nm and the best optical signal was gained in 0.2 M phosphate buffer (PBS) at pH = 6.4. The prepared NCs were applied to detect the selective enzyme activity of tyrosinase. X. Mu and co-workers also showed a new protocol using a Tyr:Au/0.75:1 molar ratio. During synthesis, a short-term heating effect was applied, which resulted in blue-emitting Tyr-AuNCs. The NCs had a 1.68% QY and were built from 10 metal atoms/cluster, as based on the mass spectrometry. They used the prepared AuNCs for selective turn-off of sensors for Fe^3+^ detection with a 0.2 μM limit of detection (LOD) [32].

Biosensing applications are a widely studied topic in NC research [11,33,34,35]. Based on the detected signal, turn-off [17,36,37] and turn-on [38,39,40] sensor systems can be distinguished by fluorescence quenching or enhancement, respectively. The identification and quantification of metal ions is a major challenge where fluorescence-based sensors have emerged as promising alternatives to expensive instrumental techniques such as inductively coupled plasma mass spectrometry, or atomic spectroscopy. For this purpose, the application of the fluorescent metal NCs as simple sensor systems can provide an excellent alternative method.

The aims of our work were focused on the development of a new synthesis method of exclusively silver-based NCs using Tyr as a reducing ligand, which has not been reported in the literature. We planned to identify the key parameters during the preparation and e comprehensive characterization was undertaken. For further application of the prepared Tyr-AgNCs as possible sensors of biologically important and harmful ions in tap water and wastewater, their interactions were investigated with several metal ions. As well as calculation of LOD values for the interesting cations, the thermodynamic parameters of the interactions were determined based on temperature-dependent and fluorescence lifetime measurements to understand the exact mechanism between the Tyr-AgNCs and metal ions.

## 2. Results and Discussion

### 2.1. Optimization of the Synthesis Protocol of the Blue-Emitting Tyr-AgNCs

For the development of new fluorescent nanomaterials, it is necessary to determine the ideal conditions by optimization of several important experimental parameters. In all cases, the aim was to reach the highest fluorescence intensity and formation of nanohybrid systems having great photostability and weak aggregation tendency.

First, the appropriate Tyr to Ag^+^ molar ratio was ascertained using a Tyr:Ag^+^/0.5:1–100:1 molar ratio, where the metal ion concentration at the start of the reaction was kept constant at 0.1 mM (Figure S1A). The preparation of the samples was made difficult by the limited water solubility of Tyr. Slight heating of the stock solution of Tyr and a small amount of NaOH was applied to increase not only the solubility but also the reduction capacity of the amino acid [41]. For preliminary synthesis, 80 °C was chosen considering the higher temperature necessary for the synthesis of noble metal NPs. Based on the experiments, it was established that weak blue fluorescence was observed in the samples after 24 h under a UV-lamp (λ_max of lamp_ = 365 nm), while a yellow color with a characteristic plasmonic band at ca. 400 nm [42] of the AgNPs did not appear. Maximum excitation and emission wavelengths were identified by fluorometric technique, which were λ_ex_ = 314 nm and λ_em_ = 408 nm, respectively. The highest intensity was detected at a Tyr:Ag^+^/5:1 molar ratio, which was applied in further experiments.

**Figure 1:**
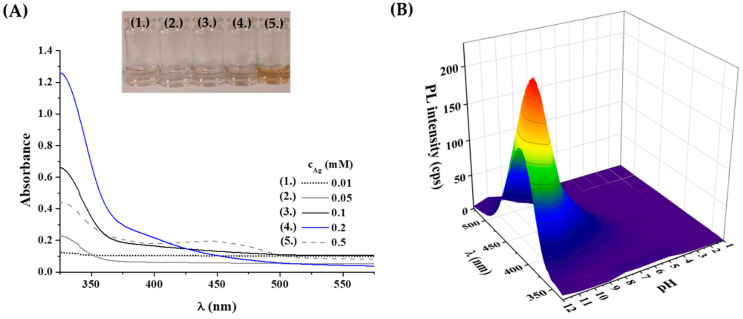
(**A**) UV-Vis spectra and photos of the prepared Tyr-Ag samples depending on the applied metal content. (**B**) Measured fluorescence spectra of the samples after 24 h synthesis time at 80 °C as a function of the applied initial pH of the reaction mixture (λ_ex_ = 320 nm; c_Ag_ = 0.2 mM; c_Tyr_ = 1 mM).

Second, the effect of metal concentration was studied using 0.01–0.5 mM Ag^+^ content. As Figure 1A represents, the UV-Vis spectra of the Tyr-Ag system show the presence of colloidal particles only when the initial metal ion concentration reaches 0.5 mM, which is confirmed by the yellowish color of the sample and the detection of a weak plasmonic band at 450 nm. Based on this observation, a lower concentration of 0.2 mM was applied for further studies.

It is well-known that the formation of ultra-small fluorescent metal dots strongly depends on the initial pH of the reaction mixture due to the pH-dependent coordination and reduction capacity of Tyr. For this purpose, the total pH range was investigated between pH = 1.0–12.0. Based on the recorded fluorescence spectra (Figure 1B), it was observed that no fluorescence could be detected under acidic conditions, perhaps due to the protonation of amino and phenolate functional groups and weak reducing capacity of Tyr. Above pH 6.0, a blue emission was observed, which showed a continuous increase to pH 11.5. At pH 12.0, a slight decrease was found. Consequently, a pH of 11.5 was selected as the ideal pH for synthesis, where the functional groups of the Tyr are all deprotonated [43] and the reducing ability is stronger [44].

Finally, the optimal temperature and the synthesis time were identified. The syntheses were carried out at five different temperatures (4; 25, 40, 60, and 80 °C), and the application of 40 °C provided products with the highest PL intensity (Figure S1B). At 4 °C, the samples did not show any fluorescence signal at all, while when using 25 °C a moderate blue emission was observed. Temperatures of 60 °C and 80 °C resulted in fast aggregation of the formed fluorescent particles. Moreover, the formation of Ag_2_O was also preferred at these higher temperatures based on the appearance of a blackish brown precipitate in the reaction mixtures.

Finally, the reaction time was also determined, using Tyr:Ag^+^/5:1; c_Ag+_ = 0.2 mM; pH = 11.5 and T = 40 °C. The emission band at 408 nm was continuously monitored by fluorometric measurements for a few days. It was observed that this characteristic band could be detected after 24 h, but showed continuous growth for 7 days (Figure S1C). Based on these data, it can be presumed that the primer sub-nanometer-sized fluorescent cores were formed within one day, but most probably the self-assembly of larger cluster aggregates with looser composition and increased luminescence needed more time (ca. 1 week) [45].

For exact structural characterization, purification of the formed nanohybrid system is necessary; therefore, a two-step method was developed. First, the non-fluorescent larger AgNPs and Ag_2_O aggregates, which appeared in the system after few days, were removed by ultracentrifugation, where the fluorescent product was in the supernatant. Thereafter, dialysis was applied by using the Pur-A-Lyzer^®^ dialysis kit, having a 1 kDa cut-off to eliminate the non-reacted metal ions and the alkali residues, for 210 min. The final metal content of the purified Tyr-Ag liquid sample was ca. 0.15 mM.

### 2.2. Optical and Structural Analysis of Tyr-AgNCs

**Figure 2:**
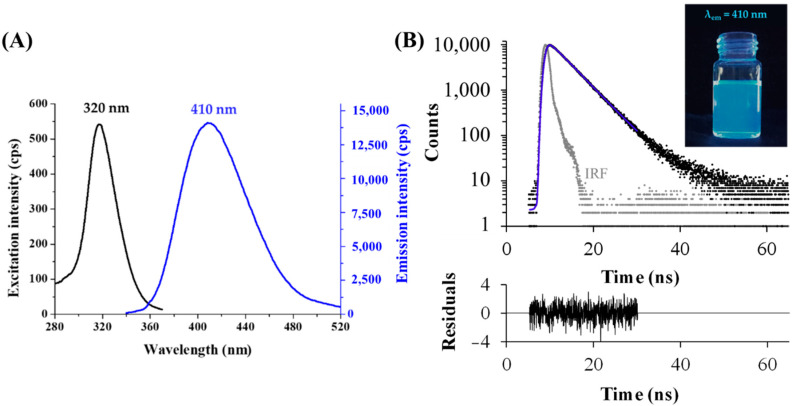
(**A**) Fluorescence excitation and emission spectra of the purified Tyr-AgNCs (T = 25 °C, c_Ag_ = 10 µM). (**B**) Fluorescence decay profile (measured: black dots, fitting: blue line, instrument response function (IRF): grey dots; λ_ex_ = 295 nm, λ_em_ = 410 nm, χ^2^ = 1.09) with the photo of the Tyr-AgNCs under UV-lamp (λ_max of lamp_ = 365 nm).

After the development of reproducible synthesis and purification protocols, the photophysical characteristic of the fluorescent Tyr-Ag nanoobjects were investigated. The samples showed blue emission under UV-lamp, while the exact excitation and emission wavelength were identified with a larger Stoke shift between both maxima at 320 and 410 nm, respectively (Figure 2A). The absolute internal quantum yield (QY%) was 1.3 ± 0.2%. Using a time-correlated single photon counting (TCSPC) method, two dominant lifetime components were obtained. Based on the registered decay curve (Figure 2B), the average lifetime was 4.28 ns, which could be separated to two main components, τ_1_ = 3.66 ± 0.09 ns (α_1_ = 50%) and τ_2_ = 4.90 ± 0.04 ns (α_2_ = 50%). The order of magnitude of the lifetime values are in good agreement with previous data [46,47], which refers to the formation of metallic NCs. Based on the extent of the Stokes shift and large full width at half maximum (FWHM) value, K. Zhang and co-workers [48] suggested that that the shorter component perhaps belongs to the metal-centered emission, while the longer component could originate from the local interaction (overlapping of p-orbitals) of the adjacent surface ligands.

**Figure 3:**
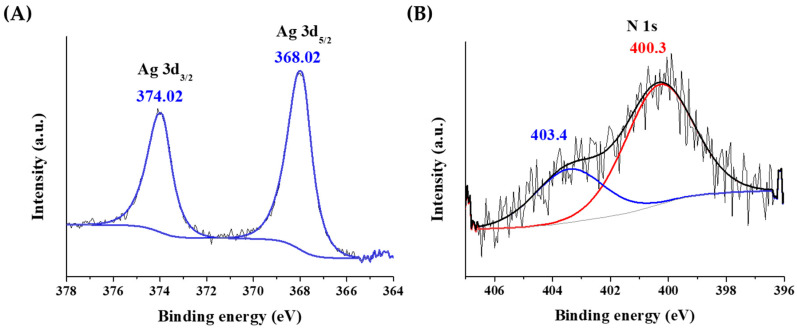
The X-ray photoelectron spectra of the (**A**) silver and (**B**) nitrogen atoms in Tyr-AgNCs.

The oxidation state of Ag was investigated by X-ray photoelectron spectroscopy (XPS). The sample was deposited on a gold substrate to avoid the overlapping of the silver peaks with other metals. As Figure 3A confirms, the asymmetric peaks of were detected. It is well-known that the location of the metallic peaks slightly depends on the size of particle cores for Ag. The peak shape extracted for the fitting of an Ag foil perfectly fitted the observed doublet. The 3d_5/2_ peak was identified at 368.02 eV, which is in good agreement with the metallic Ag peak at ca. 368.20 eV. The FWHM of the 3d_5/2_ peak was ca. 1.2 eV, which corresponds to nanocluster structure with ultra-small metallic cores [49].

In Figure 3B, the different oxidation states of nitrogen can be also observed. One form at 400.3 eV might come from the amino groups at 399.4 ± 0.8 eV. The other form is at 403.4 eV, which belongs to an oxidized form of nitrogen due to the redox reaction taking place between the tyrosine ligand and Ag^+^ ions.

To prove the direct binding between the Tyr and Ag on the cluster surface, IR studies were carried out, where the reference was the spectrum of the pure amino acid powder lyophilized under the same conditions. By comparison of the two spectra (Figure S2), the most identifiable vibration did not shift after cluster synthesis. However, significant changes were observed in e intensities, which may be due to different selection rules at the proximity of the metallic surface. In the IR spectrum of the NCs, the signals of the phenolic side chain of the amino acid and the N-terminus were strengthened, but the signals of the C-terminus were significantly weakened. The phenolic molecular part and the amino group were close to the silver cores. In the case of amino acid-stabilized noble metal NPs, it is generally accepted that the reducing effect of the Tyr is stronger under alkali conditions. According to the assumed reaction, the anionic phenolate can reduce the Ag^+^-ion by electron transfer, and thereafter the molecule shows a phenol-dienone rearrangement [50]. After particle synthesis, the stretching vibration of the carbonyl group of the new dienone molecule should appear at ca. 1670 cm^−1^. The new absorbance of the dienone should also be detected at 285–290 nm in the UV-Vis absorption spectrum. In contrast, these bands could not be observed on the IR and UV-Vis spectra of the newly synthesized Tyr-AgNCs, which proved the following formation mechanism.

A hypothesis is that phenolate reduces the Ag^+^-ion by electron transfer, while it becomes a radical fragment; two radical fragments form a dityrosine, which can stabilize the formed Ag cores [51]. The enzymatic polymerization mechanism of the Tyr-based molecules is a well-known chemical process [51,52], and the oligomerization of the amino acid has been proven by mass spectrometry in the case of nanocluster synthesis [20,21]. The vibration of the aromatic rings in the dimer amino acids are the same, and they are separated by only one bond. The ultrashort distance between the rings can cause π···π stacking of the neighboring aromatic ring, by which a significant increase of a vibration band can appear between 1540–1340 cm^−1^. It is also assumed that the dimer is linked to the metal cores through the amino groups [50].

Considering the application of the Tyr-AgNCs in bioanalytical fields, the examination of the aggregation processes was also necessary. For this purpose, the hydrodynamic diameter (d_H_), the ζ-potentials and the fluorescence properties were also monitored in the case of individual samples between pH values of 1.0 and 12.0 with constant ionic strength (*I* = 0.05 M NaCl). As can be seen on Figure S3A, the Tyr-AgNCs show great stability between pH values of 6.0 to 11.0 because the measured d_H_ < 10 nm and the ζ-potential is ca. −30 mV. Under physiological conditions (pH = 7.4, c_NaCl_ = 0.15 M) the NCs have d_H_ = 6.3 ± 2.8 nm average size and the ζ-potential is ca. −27 mV. At pH 5.0, the d_H_ reached the micrometer-sized range, while the ζ-potential was drastically decreased. This decline in pH, which is in good agreement with the charge neutral range of free Tyr ligand (pH = 4.6–7.7) [43], can cause the fast aggregation process of the Tyr-AgNCs. Under acidic conditions, the d_H_ and the ζ-potential values were continuously growing until pH 1.0. The fluorescence measurements showed a similar tendency depending on the pH (Figure S3B). The purified NCs under alkali conditions showed the highest fluorescence intensities, which were continuously decreasing at strong acidic pH.

Salt tolerance studies (Figure S4) clearly confirmed that the emission of the Tyr-AgNCs did not change considerably using c_NaCl_ = 0.001–2.5 M. Therefore, it can be concluded that the surface ligands have a great shielding effect against the steric and electrostatic destabilization effect of high ionic strength.

### 2.3. Interaction between the Blue-Emitting NCs and Different Metal Ions

#### 2.3.1. Analytical Evaluation of the Data

**Figure 4:**
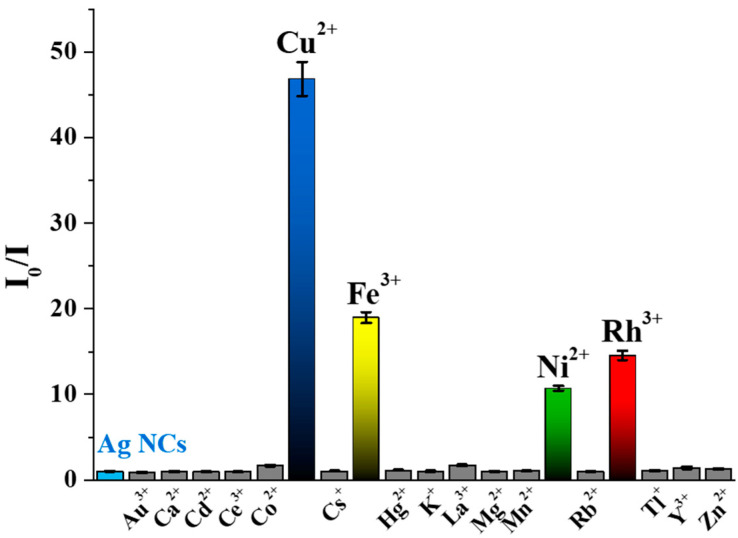
Corrected relative fluorescence of the Tyr-AgNCs in the presence of different metal ions (c_Ag_ = 18.75 µM; c_metal ions_ = 1 mM; c_NaCl_ = 1 M; T = 25 °C; λ_ex_ = 320 nm; λ_em_ = 410 nm).

To determine the usability of the initially prepared Tyr-AgNCs as a potential optical sensor for detection of metal ions in aqueous medium, the fluorescence signal was recorded before after the addition of several metal cations that can be found in tap water and wastewater. As Figure 4 shows, four different metal ions reduced the fluorescence of Tyr-AgNCs to a great extent.

Enhancement could not be observed in either case. In the calculation of the relative fluorescence (*I*_0_**/***I*), the *I*_0_ and *I* values are the corrected intensities before and after the addition of metal ions at 1 mM concentration. It was found that Cu^2+^, Ni^2+^, Fe^3+^, and Rh^3+^ ions caused ca. 50, 14, 20 and 18-fold decreases in the fluorescence of Tyr-AgNCs, respectively. To reduce the effect of ionic strength changes by the metal ions, 1 M sodium chloride was applied to keep a nearly constant ionic strength, which had no significant effect on the stability of the NCs (Figure S4).

For further analysis, calculations of the limit of detection (LOD) and dynamic range were necessary for all four ions. For this purpose, the fluorescence was measured in individual samples before and after addition of cations in the concentration range of 10 nM–5 mM. Based on the generally accepted calculation method of H. P. Lock and P. Wetzell [53], the LOD was estimated in the case of the four metal ions (Table 1). These values refer to the smallest amount of metal ions that can be identified by fluorometric measurements in aqueous samples by the quenching of blue fluorescence originating from Tyr-AgNCs dispersion. LOD values in Table 1 clearly show that the Tyr-AgNCs had different interactions with the Cu^2+^, Ni^2+^, Fe^3+,^ and Rh^3+^ ions.

Besides the LOD, the dynamic ranges were also determined, where the extent of the quenching was nearly linear with the applied concentration of the selected metal ions. Exclusive selectivity could not be determined. The EU’s drinking water standards for metal ions are Cu^2+^: 2.0 mg/L (31.5 µM); Ni^2+^: 0.02 mg/L (0.34 µM); Fe^3+^: 0.2 mg/L (3.58 µM); Rh^3+^: no data available. Taking into consideration the LOD values, if tap water contains several metal ions (Ni, Fe, Zn, Hg, Ca, Mg, K, Cd, Co) in the permitted concentration, the PL of the clusters does not change. The quenching of the clusters occurs exclusively when the Cu content of the tap water or wastewater is higher than 2 µm, so the present sensing media had a good selectivity for Cu^2+^ only.

#### 2.3.2. Physicochemical Interpretation of Interactions

For analysis of the interactions between the Cu^2+^, Ni^2+^, Fe^3+^, and Rh^3+^ ions and Tyr-AgNCs, the simple well-known Stern-Volmer fitting (Equation (1)) was carried out.

(1) $$\frac{I_{0}}{I}=1+K_{SV}\times \left[Q\right]$$

where the *I*_0_ and *I* are the corrected intensities before and after the addition of metal ions a [*Q*] mol × dm^3^ equilibrium concentration (the analytical concentration is used for the calculation in practice). The *K_SV_* is the Stern-Volmer quenching constant, which can be used as a binding constant if a static quenching mechanism applies in the studied system (vide infra) [54].

**Figure 5:**
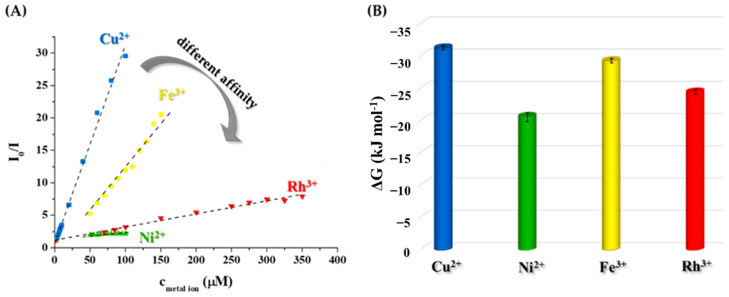
(**A**) Average relative fluorescence intensities of the Tyr-AgNCs after the addition of metal ions in the determined dynamic ranges at 25 °C (c_Ag_ = 18.75 µM, λ_ex_ = 320 nm, λ_em_ = 410 nm, *I* = 1 M by NaCl). (**B**) Calculated ΔG values of the interaction between metal ions and NCs at 298 K.

Figure 5A shows the different behavior of the Tyr-AgNCs with metal ions using the Stern-Volmer analysis. To identify the nature of the quenching, temperature-dependent measurements were carried out. Based on the change of *K_SV_* values with temperature, static and dynamic quenching could be separated. During static quenching, a non-fluorescent complex is formed between the fluorophore and the quencher, while in dynamic quenching, relaxation by a collision occurs. If the Stern-Volmer analysis shows deflection both quenching processes take place at the same time. In our work, the *K_SV_* values showed linearity with the different concentrations of metal ions in the dynamic range (Figure S5). Moreover, the determined constants decreased with the increase in temperature (Table 2) in the case of Cu^2+^, Fe^3+^, and Rh^3+^, which implies static quenching and formation of non-fluorescent complexes. During the analysis of the interaction between Ni^2+^ and Tyr-AgNCs, determined *K_SV_* values were very similar at each temperature.

To prove these assumptions, fluorescence lifetime measurements were carried out to examine the change of the two dominant lifetime components in the presence and absence of chosen cations. Under experimental conditions, the original lifetimes (τ_1_ = 3.66 ns and τ_2_ = 4.90 ns) became slightly elongated to 3.80 ns and 5.88 ns, respectively, most probably due to the higher ionic strength applied. After the addition of the metal ions to the Tyr-AgNCs, it was observed that the lifetimes did not change considerably (Table S1). No significant shortening was observed in any of the cases, which proves that generally static quenching took place between the NCs and the metal ions. A slight increase of the longer component was detected in the case of Cu^2+^ and Fe^3+^ ions. For evaluation of the different quenching ability of the studied metal ions, the coordination capability of Tyr towards the examined metal ions should be also considered. Noteworthy is that the purified NCs sample possibly could contain remnants of non-reacted Tyr ligand. At the same time, the primary quenching curves recorded (see an example in Figure S6) demonstrated a saturation-like curvature without any breakpoint or sigmoidal shape. Namely, a direct interaction between the metal ion and the Tyr-AgNCs is assumed without any side reaction (e.g., complexation of the metal ion by free Tyr ligands). Surface-exposed Tyr ligands of the NCs can possess accessible functional groups to bind the metal ions. Therefore, comparison of the stability of Tyr-metal ion complexes, which are well known for Cu^2+^ and Ni^2+^ ions in the literature, may be useful for the interpretation of the quenching trend obtained [55]. (To the best of our knowledge, for Fe^3+^-Tyr and Rh^3+^-Tyr systems no stability data are available in the literature). Concentration distribution curves in Figure S7 calculated for conditions relevant in the studied systems (c_Tyr_ = 18.75 µM, c_metal ion_ = 50 µM) reveal higher stability of the forming Cu^2+^ complexes at pH 9.0 in contrast to the Ni^2+^ complexes (0% vs. 14% free Tyr). A similar stability trend might apply when the metal ions interact with Tyr-AgNCs, this tendency shows good agreement with the quenching ability of the Cu^2+^ and Ni^2+^ ions.

To deeper understand the exact mechanism, it is necessary to determine the thermodynamics of the nanocluster/cation interactions. Based on the generally accepted approach, the Δ*G* values of the quenching processes can be calculated based on *K*_*SV*_ values using Equation (2):

(2) $$\Delta G=-RT\ln K_{SV}$$

where the Δ*G* is the Gibbs free energy change of the quenching process at *T* absolute temperature, *R* is the gas constant (8.314 J mol^−1^ K^−1^) and *K_SV_* is interpreted as conditional stability constant. 

The sign and magnitude of ΔG values can indicate the thermodynamic nature and the strength of the interaction with the selected metal ions. As can be seen in Figure 5B, the negative sign of the ΔG proves that the interaction between the metal ions and Tyr-AgNCs is thermodynamically favorable and spontaneous. The magnitudes of the Gibbs energy are ca. −31.3 kJ mol^−1^; −20.6 kJ mol^−1^; −29.4 kJ mol^−1^ and −24.5 kJ mol^−1^ at 298 K in the case of Cu^2+^, Ni^2+^, Fe^3+^, and Rh^3+^ ions, respectively. If the values are compared to each other, the affinity of Tyr-AgNCs towards the studied metal ions is the following: Cu^2+^ > Fe^3+^ > Rh^3+^ > Ni^2+^. These results are in good agreement with the assumed stability trend of Cu^2+^-NCs and Ni^2+^-NCs systems (vide supra).

**Figure 6:**
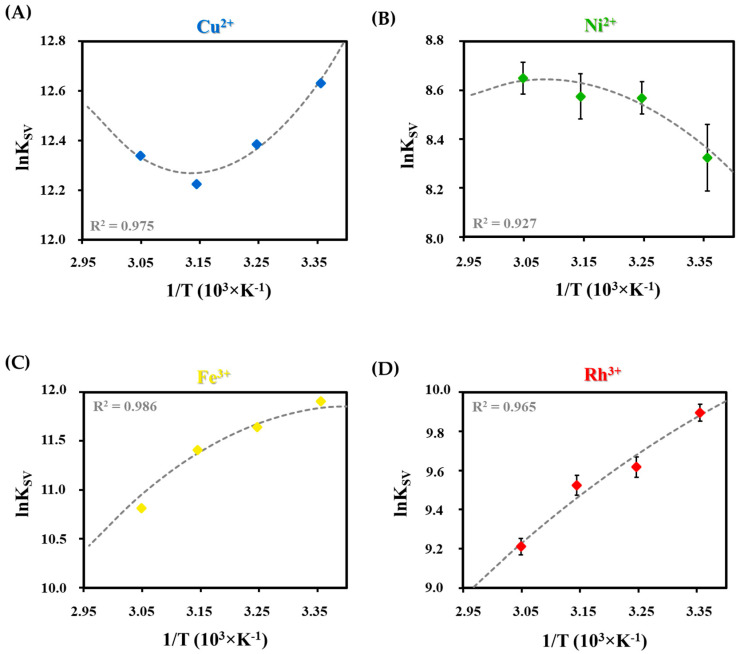
Nonlinear van’t Hoff fitting with the correlation coefficients (R^2^) in the case of (**A**) Cu^2+^, (**B**) Ni^2+^, (**C**) Fe^3+^, and (**D**) Rh^3+^ ion and Tyr-Ag NC interactions.

Based on the temperature-dependent measurements, the nonlinear regression of Van’t Hoff analysis (Equation (3)) is preferred to determine the standard reaction parameters due to the suitable correlation coefficients (*R*^2^ > 0.900) in all cases [56]:

(3) $$\ln K_{SV}=\frac{-\Delta H^{{}^{\circ}\;}\left(T^{{}^{\circ}\;}\right)}{RT}+\frac{-\Delta S^{{}^{\circ}\;}\left(T^{{}^{\circ}\;}\right)}{R}+\frac{\Delta C_{p}}{R}\;\left[\left(\frac{T-T^{{}^{\circ}}}{T}\right)-ln\frac{T}{T^{{}^{\circ}}}\right]$$

where the Δ*H°* and *ΔS°* are the standard quenching reaction enthalpy and entropy changes, respectively, *T* is the absolute temperature, *R* is the gas constant, and Δ*C_p_* is the heat capacity change. Based on the nonlinear regression, as in Figure 6, it can be concluded that the course of the fitted curves also indicates the different extent of the quenching reaction. By the sign and magnitude of the determined thermodynamic parameters, we can obtain information about the chemical forces that control the quenching reactions and the formation of the non-fluorescent complexes.

The fitted parameters are shown in Table 2. The Gibbs free energies were also calculated by Equation (4) at all temperatures based on the determined Δ*H*° and Δ*S*° to verify the accuracy of the calculations by *K_SV_* values:

(4) ΔG=ΔH°−TΔS°

where Δ*G*, Δ*H*, and Δ*S* are the Gibbs free energy, the reaction enthalpy and entropy changes of the quenching process, respectively, and *T* is the absolute temperature. Based on the results, both Δ*G* values showed good agreement with each other and proved the thermodynamically favorable and spontaneous interactions between the Ag NCs and metal ions. The previously established order in the strength of the interaction was unchanged based on the second calculation with Equation (4).

For deeper investigation of the determined values, the relation between |Δ*H*°| and |*T*Δ*S*°| indicates which plays a dominant role in the interaction between the metal ion/Tyr-AgNCs. Based on the sign and magnitude of these values, quenching-controlling forces and processes also can be identified. According to the data summarized in Table 2, the conclusions below can be made [57].

*Interaction between Tyr-AgNCs and divalent ions (Cu^2+^ and Ni^2+^):*

As a result of the spontaneous interaction of the Cu^2+^ with Tyr-AgNCs, the formation of the non-fluorescent complex is exothermic (Δ*H*° < 0). The quenching procedure of Ni^2+^ is also thermodynamically preferable via a charge neutralization process and conformation changes during the formation of the dark complex. The reaction is entropy-driven because the absolute value of the TΔ*S*° component is larger than the |Δ*H*°| in the calculated data. The positive value of the entropy change also refers to the formation of arranged solvation shells around the NCs and Cu^2+^. 

*Interaction between Tyr-AgNCs and trivalent ions (Fe^3+^ and Rh^3+^):*

The case of Fe^3+^ and Rh^3+^ ions can be discussed in the same section because the relationship between the calculated thermodynamic parameters is similar for both. Based on the data in Table 2, the spontaneous (Δ*G* < 0) complex formation is exothermic because the Δ*H*° < 0. The emergence of an ordered solvent shell is also proven by the positive values of Δ*S*°. This, perhaps, also indicates, the more dominant hydrophobic interactions between the surface ligands and presumed Fe^3+^-Tyr/Rh^3+^-Tyr complexes. In contrast to the double charged cations, the interaction with Fe^3+^ and Rh^3+^ ions are enthalpy-favored by the |Δ*H*°| > |*T*Δ°*S*| relation, which shows that intermolecular forces can facilitate the stability of the dark complex.

## 3. Materials and Methods

### 3.1. Chemicals

*L*-Tyrosine (Tyr, 99.0%), silver nitrate (AgNO_3_; 99.9%), gold(III) chloride trihydrate (HAuCl_4_; ≥99.9%), cadmium nitrate tetrahydrate (Cd(NO_3_)_3_ × 4 H_2_O; 98%), cesium chloride (CsCl; 99%), mercury(II) chloride (HgCl_2_; 99.5%), lanthanum chloride hydrate (LaCl_3_ × H_2_O; 99.9%), magnesium chloride (MgCl_2_; ≥98%), rubidium chloride (RbCl_2_; 99.8%), rhodium chloride (RhCl_3_; 98%), thallium chloride (TlCl; 98%), cerium chloride heptahydrate (CeCl_3_ × 7 H_2_O; 99.9%), yttrium(III) chloride (YCl_3_; 99.99%), cobalt(II) chloride hexahydrate (CoCl_2_ × 6 H_2_O, 98%), manganese(II) chloride tetrahydrate (MgCl_2_×4 H_2_O; 98%), nickel(II) chloride (NiCl_2_, >98%), copper(II) chloride (CuCl_2_, >98%), and iron(III) chloride hexahydrate (FeCl_3_ × 6 H_2_O, 98%) were purchased from Sigma-Aldrich (St. Louis, MO, USA). Sodium chloride (NaOH; 99.0%), hydrochloric acid (HCl; 37%), zinc(II) chloride (ZnCl_2_; 99.9%), calcium chloride dihydrate (CaCl_2_ × 2 H_2_O; 97%), potassium chloride (KCl; >99%) and sodium chloride (NaCl; 99.98%) were products of Molar Chemicals. For fresh stock solutions, Milli-Q (Merck Millipore, Burlington, MA, USA) ultrapure water (18.2 MΩ·cm at 25 °C) was used. The chemicals were of analytical grade and further purification was not applied.

### 3.2. Synthesis of Blue-Emitting Tyr-AgNCs

For synthesis, a Tyr:Ag^+^/5:1 molar ratio was applied. First, 18.2 mg Tyr amino acid was dissolved in 95 mL MQ-water, then the pH was adjusted to 11.5 using 1 M NaOH. After the dissolution of the amino acid, 5 mL of 4 mM AgNO_3_ solution was mixed into the ligand solution. The reaction mixture was thermostated at 40 °C for 7 days in the dark. At the end of the synthesis, the color changed from transparent to brownish-yellow due to a small amount of Ag_2_O and AgNPs aggregates. The purification was carried out by repeated centrifugation at 13,000 rpm for 2 h with 30 min/cycle. The prepared fluorescent NC was in the supernatant. After centrifugation, a final filtration with a 0.45 μm pore-sized hydrophilic syringe filter and dialysis for 210 min with standard cellulose membrane (cut-off = 1 kDa) were used to remove the excess metal ions, ligand, and alkali.

### 3.3. Characterization Methods

Fluorometric measurements were carried out on a JASCO FP-8500 spectrofluorometer equipped with an ETC-815 Peltier-module using 1 cm optical length (λ_ex_ = 320 nm, λ_em_ = 410 nm, bandwidth = 2.5–2.5 nm). For the determination of the absolute internal quantum yield (QY%), a JASCO ILF-835 integrating sphere (d = 100 mm) was attached to the spectrofluorometer, and the SpectraManager 2.0 Quantum yield calculation program was used. The fluorescence lifetime was measured using a Horiba Fluoromax-4 fluorometer equipped with a DeltaHub time-correlated single photon counting (TCSPC) controller using a NanoLED light source N-295 (Horiba Jobin Yvon). The emitted light was detected at λ_em_ = 410 nm with a 6 nm slit width; the number of counts on the peak channel was 10,000. The number of channels used for the analysis was ca. 1000, with a time calibration of 0.02532 ns/channel. For the registration of the instrument response function, Ludox^®^ (from Sigma-Aldrich) was used as a scatter solution. Further background correction was not needed. The program DAS6 (version 6.6.; Horiba Jobin Yvon, Paris, French) was used for the analysis of the experimental fluorescence decays. A JASCO V-770 Spectrophotometer was used to record the UV-Vis spectra between 190–800 nm with a 1 cm optical path length. Fourier transform infrared (FT-IR) spectroscopy studies were performed using a BIO-RAD Digilab Division FTS-65A/896 Fourier Transform infrared spectrometer with a Harrick’s Meridian^®^ SplitPea single-reflection diamond attenuated total reflectance (ATR) accessory. All IR spectra were recorded at a 4 cm^−1^ optical resolution by averaging 256 interferograms. For the studies of the oxidation state of the metal content and the heteroatoms of the ligand molecule, X-ray photoelectron spectroscopy (XPS) measurements were used. The instrument was a SPECS instrument equipped with a PHOIBOS 150MCD9 hemispherical analyzer, under a main-chamber pressure in the 10^−9^–10^−10^ mbar range. The fixed analyzer transmission mode was applied with 40 eV pass energy for the survey scans and 20 eV pass energy for the high-resolution scans. The sample powder was deposited on gold foil by multistep cyclic freeze-drying. The Al Kα X-ray source was used at 200 W power. Charge referencing was done to the 4f peak of the platinum substrate (71.00 eV) on the surface of the sample. For spectrum evaluation, CasaXPS commercial software package was used. The hydrodynamic diameters were calculated by the Smoluchowski model and measured together with the ζ-potentials on a Malvern Zetasizer NanoZS ZEN 4003 apparatus equipped with a He-Ne laser (λ = 633 nm) at 25 ± 0.1 °C and 0.1 M ionic strength.

### 3.4. Measurement of the Interactions between Metal Ions and Tyr-AgNCs

Due to the strong absorption of the transition metals and to avoid the false interpretation of selectivity, a correction of the registered spectra was carried out according to the following equation (Equation (5)) [54]:

(5) $$I_{corr}=I_{m}\times 10^{\left(A_{EX}+A_{EM}\right)/2}$$

where the *I_corr_* is the corrected fluorescence intensity, and the *I_m_* is the measured intensity at 410 nm. *A_EX_* and the *A_EM_* refer to the absorbance at the excitation wavelength at λ_ex_ = 320 nm and absorbance at the emission wavelength at λ_em_ = 410 nm. For the evaluation of fluorescence quenching data, the *I_corr_* values are indicated as *I* values for clarity in this work. 

The applied final volume was 2 mL in the case of each individual sample. For this purpose, the following procedure was applied. The pH of 250 µL of Tyr-AgNCs was adjusted to 9.0 and it was mixed with 1 mL (pH = 9.0) of sodium chloride solution to reach a high constant 1 M ionic strength. To identify the effect of metal ions, the 250 µL of metal ion solution was added into the sample, where the final concentration was 1 mM. To study the quenching effect of Cu^2+^, Ni^2+^, Fe^3+^ and Rh^3+^, the sensor probes were designed similarly, but the tested final concentrations of the cations were varied between 10 nM and 5 mM. The sensor measurements were done using a Horiba Jobin Yvon Fluoromax-4 spectrofluorometer with a 1 cm optical length. The excitation wavelength was 320 nm with a 3 nm slit widths The samples were thermostated in a water jacketed quartz cuvette.

## 4. Conclusions

We developed a reproducible procedure for the synthesis of blue-emitting Tyr-AgNCs. For this purpose, several parameters such as molar ratios, initial pH, metal content, temperature, and synthesis time were optimized to reach the maximal fluorescence intensity. To clean the suspension, a cyclic ultracentrifuge method was combined with the last dialysis with a standard cellulose membrane having a 1 kDa cut-off. Based on exact structural characterization, it was concluded that the formed nano-object was a nanocluster-based system, which showed blue emission with maximum intensity at 410 nm. The clusters contained metallic cores confirmed by XPS, which are stabilized by Tyr amino acid through the amino groups of dityrosine molecules. The clusters showed great stability between pH values of 6 and 11, proven by measured hydrodynamic diameters (d_H_ < 10 nm) and ζ-potential values (*ca.* −28 mV). The PL properties were in good agreement with these data because the fluorescence continually decreased below pH 6.0.

In order to use our newly produced Tyr-AgNCs in the design of selective optical sensors for detection of different di- or trivalent metal ions, several experiments were carried out to identify the limitations and possibilities of our sample. Based on tested metal ions, it was shown that four ions (Cu^2+^, Ni^2+^, Fe^3+^, and Rh^3+^) had an effect on the fluorescence property of the Tyr-AgNCs. However, exclusive selectivity could not be determined, but different affinities were observed towards these ions. Applying a generally accepted method, the limits of detection, as well as the dynamic ranges for the detection of the Cu^2+^, Ni^2+^, Fe^3+^, and Rh^3+^ ions were determined. To understand the differences between the interactions, temperature-dependent and time-correlated fluorescence measurements were conducted. Based on the result, it was determined that static quenching takes place predominantly. By Stern-Volmer analysis, quenching constants were calculated and applied for further physicochemical evaluation of the data. The effects are ranked as follows: Cu^2+^ > Fe^3+^ > Rh^3+^ > Ni^2+^ based on the calculated ΔG values. The determined thermodynamic parameters showed the dominant forces and processes that regulate the formation of non-fluorescent complexes between Tyr-AgNCs and the studied metal ions.

## Figures and Tables

**Table 1:** Calculated LOD and dynamic range values of Cu^2+^, Ni^2+^, Fe^3+^ and Rh^3+^ ions.

Type of Metal Ion	Limit of Detection (μM)	Dynamic Range (μM)
Cu^2+^	2.07 ± 0.18	2.5–100
Ni^2+^	49.12 ± 1.3	50–100
Fe^3+^	48.28 ± 0.7	50–150
Rh^3+^	66.65 ± 0.2	70–350

**Table 2:** Fitted Stern-Volmer constant (*K_SV_*) of the interaction between Tyr-AgNCs and Cu^2+^, Ni^2+^, Fe^3+^ and Rh^3+^ ions with the correlation coefficients (*R*^2^), the calculated Δ*G* values calculated by Equations (3) and (5), the standard enthalpy (Δ*H****°***) and standard entropy (Δ*S°*) changes by the nonlinear Van’t Hoff regression at four different temperatures (*T*).

Type of Metal Ion	*T* (K)	*K_SV_* (M^−1^)	*R* ^2^	Δ*G* ^i^ (kJ mol^−1^)	Δ*H*° (kJ mol^−1^)	Δ*S*° (kJ mol^−1^ K^−1^)	Δ*G* ^ii^ (kJ mol^−1^)	Δ*C*_*p*_ (kJ mol^−1^)
Cu^2+^	298	306,100 ± 6180	0.995	−31.31 ± 0.01	−14.29 ± 8.46	0.056 ± 0.025	−31.09	−1.35 ± 1.17
	308	239,050 ± 4330	0.996	−31.66 ± 0.01			−31.66	
	318	203,750 ± 4650	0.994	−32.44 ± 0.02			−32.22	
	328	228,260 ± 2830	0.998	−33.64 ± 0.01			−32.78	
Ni^2+^	298	4120 ± 850	0.920	−20.62 ± 0.06	3.72 ± 1.24	0.083 ± 0.030	−21.01	−0.07 ± 0.05
	308	5260 ± 320	0.947	−21.94 ± 0.02			−21.84	
	318	5300 ± 110	0.953	−22.67 ± 0.01			−22.67	
	328	5700 ± 370	0.943	−23.58 ± 0.02			−23.50	
Fe^3+^	298	147,960 ± 6250	0.984	−29.36 ± 0.01	−19.36 ± 6.50	0.034 ± 0.013	−29.59	1.36 ± 0.90
	308	113,610 ± 2570	0.995	−29.93 ± 0.01			−29.93	
	318	90,140 ± 1850	0.996	−30.06 ± 0.01			−30.27	
	328	49,710 ± 2680	0.975	−29.75 ± 0.02			−30.62	
Rh^3+^	298	19,850 ± 950	0.982	−24.52 ± 0.01	−15.81 ± 8.02	0.029 ± 0.014	−24.52	0.39 ± 0.05
	308	15,030 ± 690	0.984	−24.79 ± 0.01			−24.81	
	318	13,690 ± 520	0.989	−25.01 ± 0.01			−25.11	
	328	10,010 ± 310	0.993	−25.16 ± 0.01			−25.40	

^i^ calculation by $\Delta G=-RT\ln K_{SV}$. ^ii^ calculation by ΔG=ΔH°−TΔS°.

## Data Availability

The supporting raw datasets are available at the laboratory of MTA-SZTE Lendület “Momentum” Noble Metal Nanostructures Research Group. Please contact the corresponding author of the paper.

## Supplementary Materials

The following supporting information can be downloaded at: https://www.mdpi.com/article/10.3390/ijms23179775/s1.
