# Supplementary material for: Fluorescence Quenching of Tyrosine-Ag Nanoclusters by Metal Ions: Analytical and Physicochemical Assessment
Source: Int J Mol Sci. 2022 Aug 29;23(17):9775. doi: 10.3390/ijms23179775 (PMC9456322; doi:10.3390/ijms23179775)
Supplement: Supplementary file 1 [file ijms-23-09775-s001.zip › ijms-1872520-supplementary.pdf]

SUPPORTING INFORMATION

Fluorescence Quenching of Tyrosine-Ag Nanoclusters by Metal Ions: Analytical and Physicochemical Assessment

Ditta Ungor [1,2], Rita Bélteki [1,2], Krisztián Horváth [2], Orsolya Dömötör [3], and Edit Csapó [1,2,*]

[1] MTA-SZTE Lendület "Momentum" Noble Metal Nanostructures Research Group, University of Szeged, H-6720 Rerrich B. sqr. 1, Szeged, Hungary
[2] Interdisciplinary Excellence Center, Department of Physical Chemistry and Materials Science, University of Szeged, H-6720 Rerrich B. sqr. 1, Szeged, Hungary
[3] Department of Inorganic and Analytical Chemistry, University of Szeged, H-6720 Dóm sqr. 7, Szeged, Hungary
* Correspondence: E. Csapó: juhaszne@chem.u-szeged.hu

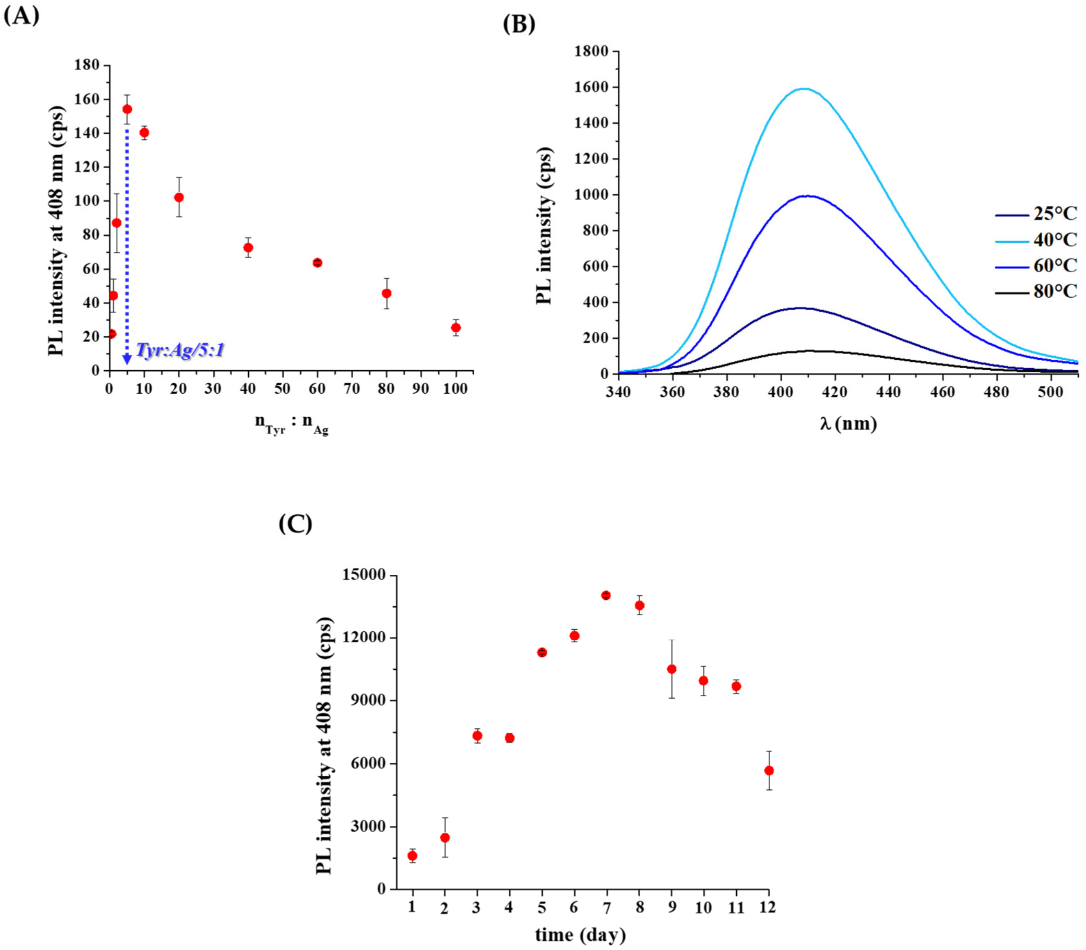

Figure S1. (A) The emission intensity at 408 nm depending on the applied molar ratios of Tyr:Ag^+ system after 24 h (λ_{ex} = 314 nm; c_{Ag} = 0.1 mM; $T_{reaction}$ = 80 °C; pH = 12). **(B)** The emission spectra of the samples depending on the applied reaction temperature after 24 h (Tyr:Ag^+/5:1; λ_{ex} = 314 nm; c_{Ag} = 0.2 mM; pH = 11.5). **(C)** The emission intensity values at 408 nm depending on the reaction time (Tyr:Ag^+/5:1; λ_{ex} = 314 nm; c_{Ag} = 0.2 mM; pH = 11.5; T = 40 °C).

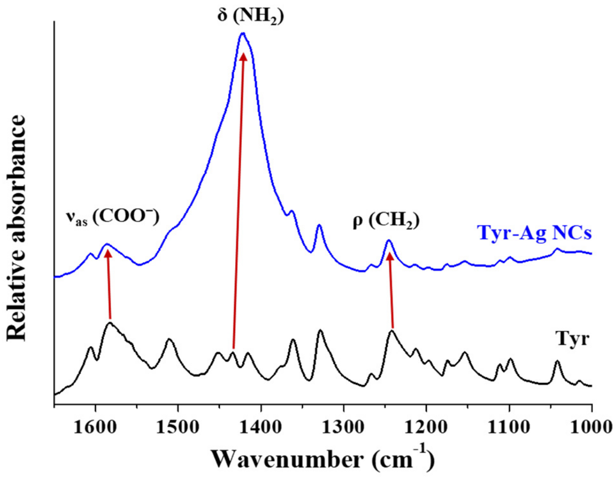

Figure S2. The IR spectra of the pure amino acid (black line) and the prepared Tyr-AgNCs (blue line).

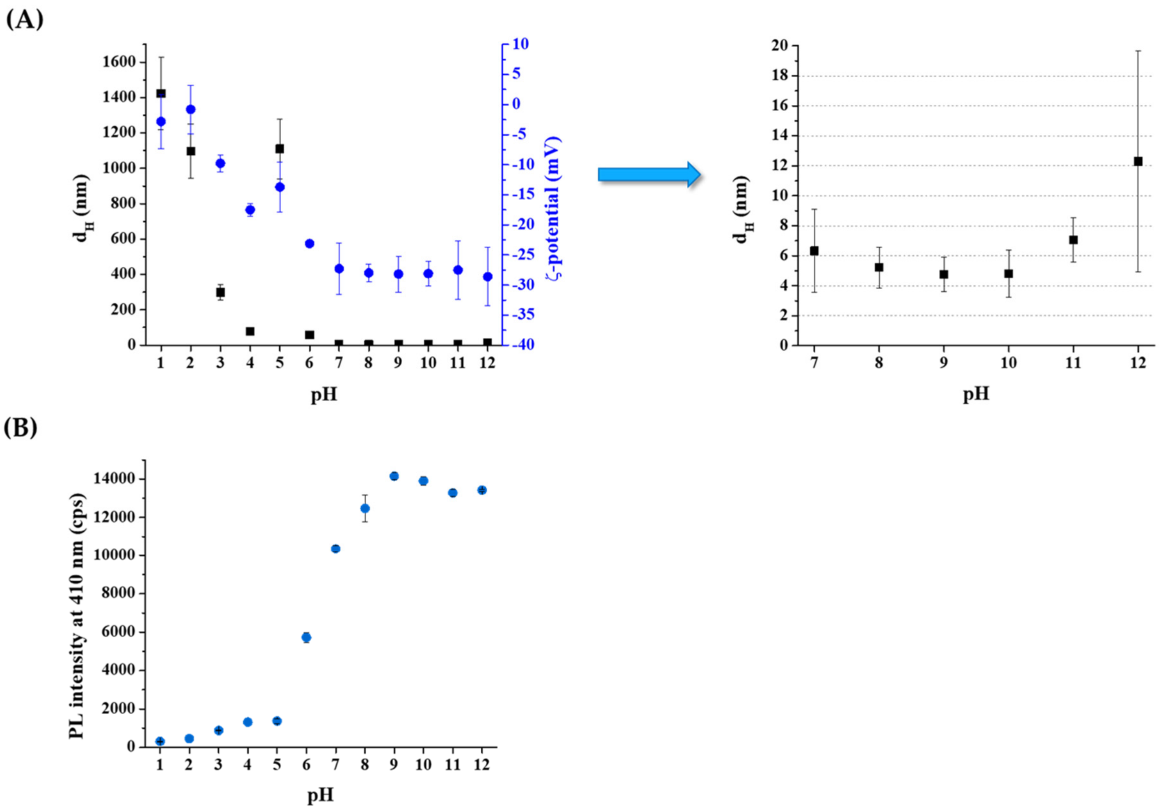

Figure S3. (A) The measured hydrodynamic diameters (d_H) and the ζ-potentials of the Tyr-AgNCs depending on the pH with the set of the diameters between pH = 7–12 (I = 0.05 M by NaCl). **(B)** The emission intensity of the Tyr-AgNCs depending on the pH of the purified NCs dispersion (λ_{ex} = 320 nm; λ_{em} = 410 nm; T = 25 °C; I = 0.05 M by NaCl).

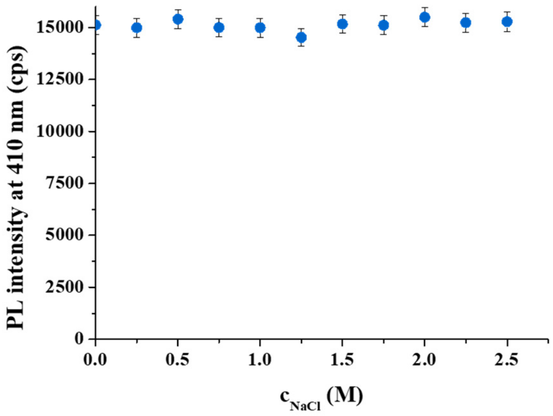

Figure S4. The detected emission intensity of the Tyr-AgNCs at different concentration of NaCl (λ_{ex} = 320 nm; λ_{em} = 410 nm; T = 25 °C; pH = 11).

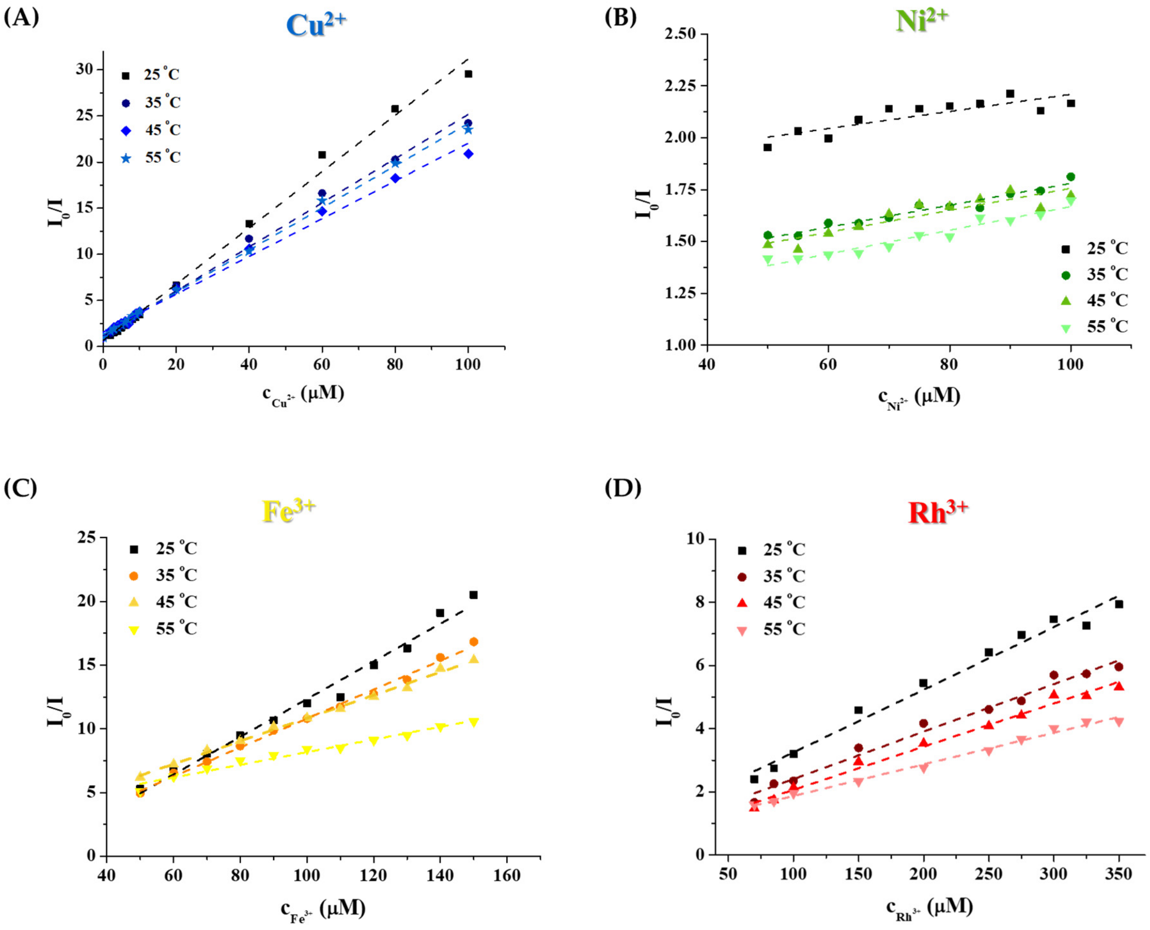

Figure S5. The Stern-Volmer fitting of the quenching process in the case of **(A)** Cu^{2+}, **(B)** Ni^{2+}, **(C)** Fe^{3+}, and **(D)** Rh^{3+} ion at different temperatures.

Table S1. The determined lifetimes (τ) before and after addition of Cu^{2+}, Ni^{2+}, Fe^{3+}, and Rh^{3+} ions (T = 25 °C; c_{NaCl} = 1 M; pH = 9.0; λ_{ex} = 295 nm; λ_{em} = 410 nm, c_{Ag} = 187.5 μM).

	Tyr-AgNCs without metal ion	Cu^{2} 60 μM^{+}	Ni^{2+} 60 μM	Fe^{3+} 60 μM	Rh^{3+} 100 μM
τ_1 (ns)	3.80 ± 0.03	3.76 ± 0.07	3.40 ± 0.07	3.37 ± 0.05	3.30 ± 0.07
τ_2 (ns)	5.88 ± 0.03	6.16 ±0.03	5.65 ± 0.02	6.50 ± 0.02	5.79 ± 0.02
χ^2	1.09	1.14	1.06	1.26	1.14

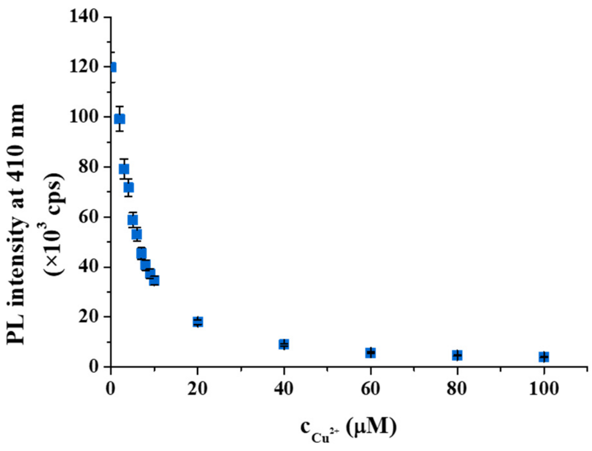

Figure S6. Representative quenching curves for the **(A)** Tyr-AgNCs – Cu^{2+} systems (T = 25 °C; c_{NaCl} = 1 M; pH = 9.0; λ_{ex} = 295 nm; λ_{em} = 410 nm, c_{Ag} = 18.75 μM).

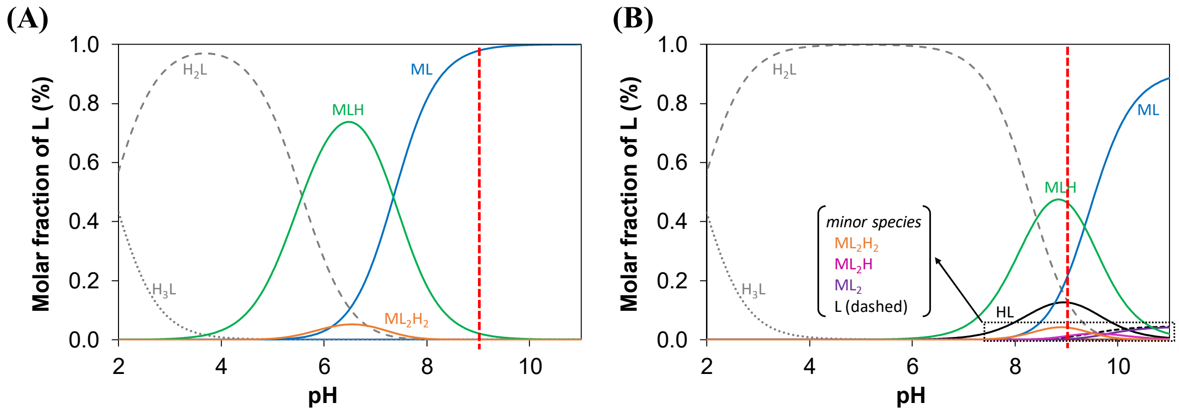

Figure S7. Concentration distribution curves of the **(A)** Cu^{2+}:Tyr (1:0.38) and **(B)** Ni^{2+}:Tyr (1:0.38) systems, calculated on the basis of stability constants reported by L.D. Petit et al. Ref. [55]. *L* denotes the completely deprotonated Tyr, charges of the species are omitted for simplicity. ($c_{metal\ ion}$ = 50 μM; c_{Tyr} = 18.75 μM; I = 0.1 M (KNO_3); T = 25 °C)
Note: concentration of the Tyr corresponds to that of the NCs applied in the quenching experiments.
